# An Experimental Inquiry into the Function of the Supra-Renal Capsules and Their Supposed Connexion with Bronzed Skin

**Published:** 1858-03

**Authors:** Geo. Harley

**Affiliations:** University College, London


From the British and Foreign Medico Chirurgical Review.

*An Experimental Inquiry into the Function of the Supra-Renal Capsules and their supposed Connexion with Bronzed Skin.* By Geo. Harley, M.D., F.C.S., of University College, London.

There are a number of organs in the body that have very generally received the name of " ductless glands." The term gland being applied to them, not on account of their being known to possess any secretion, but simply from the fact of their microscopical structure resembling in many respects that of the ordinary secreting organs.

With regard to their functions, it may be said that nothing is as yet positively known. And it is easy to understand why this should be the case, when we remember that they possess no duct, and consequently their secretion cannot be obtained for analysis, the only direct channel by which we arrive at a knowledge of the special function of any secreting organ. It is true that other methods of investigation are open to us, but these unfortunately are the least perfect, and most circuitous. Moreover, they have frequently been employed by different observers, and invariably yielded only negative results. In this, as in every other department of medical science, much has been written upon what was least understood. We consequently find many theories in circulation regarding the functions of these organs. Take, for example, the supra-renal capsules, which are now about to occupy our attention, and we shall find that they have been at one time or other, agreeably to the fancy of the author, appended to the vascular, nervous, urinary, and sexual systems. Theorists had here ample scope for their talents, there being few facts connected with the history of these organs to lay hold of; they worked in the dark, and the result of their united labors has been the retardation instead of the advancement of science. Fortunately for the present generation, the members of the hypothetical school of medicine are fast dying out, and their ranks are being recruited from among those who can thoroughly appreciate the relative value of fact and theory. In proof of this latter statement, we need only to look at the manner in which the function of the supra-renal capsules has, both at home and abroad, been lately investigated. Instead of men seeking facts in the support of theory, theory has been made altogether subservient to fact. And although, notwithstanding this critical investigation, the function of these organs has not yet been unveiled, many important physiological and pathological facts have been added to their literature.

To Dr. Addison is due the merit of having attracted the attention to, and stimulated men to inquire into the nature of, the supra-renal bodies.

It was after the perusal of his interesting monograph that I, like others, was tempted to try to discover their function. The following paper is the result of the investigation.

In order to make the subject of my communication as concise as possible, I shall divide it into four parts, namely—

1st. The histology of the supra-renal capsules.

2d. Their chemical composition.

3d. The effects of their removal from healthy animals.

4th. Their pathology.

*Morphology of the Supra-renal Bodies.*—On making a transverse section of the supra-renal capsules, they are seen by the naked eye to be composed of two differently colored substances; the external cortical substance being of a yellow color, and sometimes marked with perpendicular striæ; the internal or medullary part, of a slate or reddish-brown hue, and pierced with large openings. These opening are the mouths of the venous sinuses. On a thin section of the cortical portion being placed under the microscope, it is seen to be composed of a large quantity of yellow colored cells, arranged in irregularly-sized rows, placed perpendicularly to the surface, and imbedded in a fibro-areolar matrix. In the human supra-renal capsule, the fibrous nature of the cortical substance, as well as the direction of the fibres, is occasionally sufficiently well marked to be visible to the naked eye on simply tearing or breaking it across.

If the individual cells constituting the rows be examined, they are seen to resemble in size and general appearance the cells lining the uriniferous tubes of the kidney, except that they usually possess a much darker colour. They consist of a homogeneous cell wall filled with variously sized granules, the greater part of which are deeply tinged with yellow pigment. The cells in general contain a considerable quantity of fat, and possess a nucleus, although it is not always visible without the application of reagents. When floating free in the field of the microscope, the cells appear irregularly round, but when grouped together in masses, they have a somewhat polygonal form. In order to study their arrangement in the fibro-areolar matrix, it is necessary to employ very fine sections of the capsules, which, however, are difficult to prepare from the fresh human supra-renal body, especially if it contains much fatty matter. It is therefore advisable to harden the substance of the capsule by keeping it during a few days in a weak solution of chromic acid. After which treatment it will usually admit of being cut sufficiently thin for examination

Considerable difference of opinion exists among histologists regarding the manner in which the cells of the cortical substance are arranged.—Mr. Simmons thinks that the columnar masses which they form are a series of closed tubes lying perpendicular to the surface of the organ; while Kölliker, Ecker, Frey, and most other observers describe the cells as being grouped together in a number of oblong vesicles, lying in parallel row, but not communicating with each other. Mr. Gray, on the other hand, believes that the adjoining walls of the vesicles are sometimes removed by absorption, and thus give rise to tubular cavities.

My own opinion is, that the cells in the cortical substance of the human supra-renal bodies are grouped together in masses of various lengths, and arranged in the form of rows, which gives to them the appearance of parallel tubes. The sacculi enclosing the cell-masses vary in breadth; sometimes they are wide enough to admit of two, or even three cells lying abreast of each other within them. Occasionally, however, their diameter is only sufficiently to admit of one cell. When they are wide they are usually very short, looking more like round vesicles. When narrow they are generally long; sometimes extending through the whole depth of the cortical substance. If their transverse diameter is sufficient to admit of one cell only, they have usually a very beautiful appearance, in consequence of the single row of cells which compose them, being arranged like a single line of bricks closely set together. These columnar-shaped cell-masses are surrounded by fibro-areolar tissue, in which ramify the vessels and nerves.

As the cortical becomes blended with the medullary substance, the columnar arrangement of the cell-masses disappears, its place being taken by irregularly-sized vesicles containing from one to five cells. The vesicles, which are nothing more than short cell-masses, are unsymmetrically distributed in the medullary substance. They sometimes cease at a short distance from the edge, at other times are scattered almost throughout the whole extent of the medullary substance, from which they are easily recognised by their yellow colour.

I have here avoided using the term "tube," which was applied to the cell-masses by Simon, as I think they scarcely deserve that title. For when examined in transverse sections they are seen to present a very different appearance from that of true tubes; such, for example, as we find in the kidney. In a transverse section of the latter, the cells are seen to be symmetrically arranged on a basement membrane round an opening; while in the former, the cells are more or less irregularly distributed, without having any trace of a central cavity.

In describing the cavities containing the cells, Kölliker speaks of them as mere floculi in the stroma of the organ, possessing no basement membrane. With all due deference to the opinion of my former teacher, which I have just cause to appreciate highly, I must say that I believe Ecker and Frey are correct in stating that the loculi are flined with a homogeneous membrane. I have been able to demonstrate it in longitudinal sections of the cortical substance by carefully washing away some of the cells. The membrane, although exceedingly delicate and transparent, is, however, distinctly visible at those points where it happens to be thrown into folds.

The dark slate-coloured medullary substance has a very different appearance under the microscope from that presented by the cortical part.—When viewed by transmitted light, it is of a paler colour, and with a high magnifying power is seen to be composed of large pale nucleated cells, tolerably regularly distributed in a fibro-granular matrix. In the medullary substance of the supra-renal capsules of some ruminant animals, Mr. Gulliver has noticed large reddish-coloured corpuscles. Kölliker and some others describe the cells in the medullary substance as very closely resembling ganglion corpuscles. To my mind they have much more the appearance of secreting cells, such as we find in the liver and kidney. In describing the structure of the medullary substance of the supra-renal capsule of the land salamander, Leydig speaks of one part of it being almost entirely composed of dirty yellow ganglion corpuscles; and that these through a gradual change in their contents, pass directly into the fatty granular cells proper to the supra-renal body.

The structure of the medullary substance of the supra-renal capsules of the different animals varies very much. In the horse, Frey says that it contains, like the cortical substance, well marked gland vesicles (cell-masses). In birds the medullary is usually not distinguishable from the cortical substance. I have seen an appearance in some human supra-renal capsules similar to that said to occur in the horse, and yet the capsules appeared otherwise healthy.

Upon histological grounds, Kölliker asserts that he would regard the function of the cortical and medullary substances as perfectly distinct from each other. The former part he looks upon as being closely allied to a secreting organ, probably in some way connected with the vascular system; while the latter, in consequence of its richness in nerves, and its containing cells resembling ganglion corpuscles, he regards as an apparatus connected with the nervous system. Leydig and Bergmann go a step farther, and say that the supra-renal capsules stand in so close a relationship with the nervous system, that they ought to be considered as part of it.

*Are the Supra-renal. Capsules Fœtal Organs?*—The supra-renal capsules have generally been regarded as organs more particularly belonging to fœtal life. Remak says that they are developed simultaneously with, but independently of, the kidneys, and that they are originally larger than the latter organs. Most writers state that they diminish after birth, both in relative size and in activity of function, with advancing years. This is, however, only true to a certain extent; for although the supra-renal capsules do not bear the same relation to tne size of the adult as they do to that of the fœtus, yet their growth, when compared with that of some of the other internal organs, is very much greater than has been usually supposed.

According to Meckel, in the human embryo, at the third month, the supra-renal capsules and the kindeys are of equal size. At the sixth month the former are to the latter as 2 to 5, and at the ninth month as 1 to 3; whereas after birth the proportion is in the child as 1 to 8, and in the adult as 1 to 28.* These figures certainly speak strongly in favor of the view that the human supra-renal bodies are organs whose function is chiefly performed during fœtal life; but they prove nothing more. On the other hand, the observations that have been made upon these organs in animals by Ecker, Frey, Brown-Séquard, and myself, tend to prove that their function is carried on with considerable activity during adult age. Frey states, that in certain mammalia the relative weight of the supra-renal capsules and kidneys is the same in the adult as in the embryo. Ecker makes a similar remark with regard to the *coluber natrix*. And Brown-Séqnard says, that in dogs, cats, and guinea pigs, the weight of the capsules after birth increases in the same, if not perhaps in a greater, ratio than that of the kidneys. My own observations have been limited to one species of animal—the cat—and are far from being numerous, having only been made upon thirteen fœtal capsules and six adult capsules. But I think they are sufficiently interesting to be here quoted.

I found that five capsules from fœtal kittens, which appeared to be more than two-thirds developed, weight 0.03 grammes; while five kidneys from the same fœtuses weighed 0-31 grammes, making the relative weight of the former to that of the latter, as 1 to $10\frac{1}{10}$. On the other hand, the two supra-renal capsules from the mother of the kittens weighed 0.502 grammes, and the two kidneys 19.167 grammes, thus making the weight of the adult capsule to that of the kidney as 1 to 28.18. On another occasion I found that eight supra-renal capsules taken from kittens immediately after birth weighed 0.110 grammes, while the eight

* Handbuch der Anatomie, Band iv. p. 507.

kidneys from the same animals weighed 6.336 grammes. Four capsules, two from the parent of the kittens, and two from another cat, weighed 0.722 grammes; while the four kidneys of the same animals weighed 39.534 grammes. The weight of the supra-renal capsule, therefore, in the kitten at birth is to that of the kidney as 1 to 57.3 ;* and in the adult, in the examples last cited, as 1 to 54.8. But as so few adult capsules and kidneys were employed, it may be perhaps better to take the average of the whole six that were examined (1 to 38.18 and 1 to 54 8) which would then give us the relative weight of these organs in the full-grown animal as 1 to 46.49.†

Now if we look upon the average weight of the supra-renal capsule of the new-born cat as being 0.0137 grammes ($\frac{0.110}{8}=0.0137$), and of the kidney as 0.792 grammes ($\frac{6.336}{8}=0.792$); while we regard the average weight of the adult capsule as 0.204) grammes ($0.722 + 0.502 : 6 = 0.204$), and that of the kidney as 9.783 grammes ($39.534 + 19.167 : 6 = 9.783$); we shall find by a very simple calculation that the kidney has after birth increased 12.35 times in weight, while the supra-renal capsule in the same time has increased 14.87 times in weight. What conclusion are we to draw from that fact? We here see that the supra-renal capsules increase in size as age advances at a greater rate than the kidneys. I must here speak with caution, however. Small statistics are dangerous data to draw conclusions from, and perhaps a larger number of observations might furnish us with different results. I think, however, that I may say without reserve that the development of the supra-renal capsules is not arrested after birth; and, moreover, as they continue to increase in size at a certain ratio, they ought not, strictly speaking, to be regarded as fœtal organs—like the thymus gland, for example. I quite agree, therefore, with Dr. Brown when he says,

"Des organes que ne s'atrophient pas, et, encore plus, des organes qui croissent d'une manière notable depuis la naissance jusqua'à l'âge adulte, sont des organes qui fonctionnent; il est donc inexact de dire que les capsules surrénales appartiennent exclusivement à la vie embryonnaire."‡

*The Chemistry of the Supra-renal Capsules.*—The chemical literature of the supra-renal capsules may be summed up in a very few words; for, as far as I am aware, there are only two authors who have attempted to give us any information on this point. In the beginning of last year,

---

* This corresponds exactly with the result obtained by Ecker, who says the relation between the kidneys and capsules in the kitten at birth is as 1 to 56 or to 60.

† The relative size of the supra-renal capsules and kidneys varies very considerably in different animals: in the seal, for example, it is as 1 to 150, and in the guinea-pig as 1 to 5. (Frey.)

‡ Archives Générales de Méd., Oct. 1856.

Monsieur Vulpian communicated to the Société de Biologie the discovery of a very peculiar reaction possessed by the supra-renal capsules of vertebrated animals. He found that an aqueous solution of iodine brought in contact with the medullary substance gave rise to a beautiful rose color. And further, that the greater portion of oxidizing agents acted like the iodine solution, although in a minor degree. Even the oxygen of the air, under the influence of light, produced the same effect. I have repeated Monsieur Vulpian's experiment on the medullary substance of the supra-renal capsules of sheep with success; but am equally at a loss with the author to form an opinion as to the nature of the substance which possesses the strange property alluded to. We as yet know so little regarding the nature of color and coloring matters, that it would be futile to attempt to draw any conclusions with regard to the import of the rose-colored reaction above spoken of.

Not being able to separate the coloring matter, Monsieur Vulpian, in concert with M. Cloez, proceeded to extract the "immediate principles" of the supra-renal bodies; and these gentlemen have recently communicated to the Institute de France as the result of their united labors, the discovery of hippuric and taurocholic acids in the supra-renal capsules of herbivorous animals.* But as these substances have been abundantly found in different parts of the animal body, their discovery in the latter organs, although interesting, unfortunately throws no light upon the nature of their functions.†

*Physiology of the Supera-renal Capsules.*—Unfortunately, we do not as yet possess much experimental research regarding the function of the super-renal capsules. It is only since the publication of Dr. Addison's monograph that physiologists have attempted to unveil the impenetrable obscurity which hangs around them, by studying the effects of their removal from the bodies of healthy animals. And I believe that Gratiolet,‡ Brown-Sequard,§ Philipaux,|| and myself,¶ are the only observers who have as yet made the result of their observations public. It unfortunately happens, too, that the fruits of our experience have led us – or I should rather say one of us—to opposite conclusions, as will appear in the following pages, where I shall attempt to reconcile some of the discrepancies.

---

* Comptes Rendus, Sept. 7th, 1857.

† Since the preceding remarks were in type, I have seen a communication by Virchow (Archiv f. pathol. Anat. Bd. xii. Hft. 4 u. 5) on the chemistry of the supra-renal capsules, in which he mentions that he has found Leucine in the medullary substance. He likewise confirms the observation of M. Vulpian with regard to the rose-colored reaction.

‡ Comptes Rendus, 1856. § Ib 1856. || Ib. 1855-7.

¶ Reports of the Pathological Society, London, 1857.

In endeavoring to ascertain by direct experiment the function of the capsules, I removed them from several different species of animal: the dog, the cat, the guinea-pig, the mouse, and the rat. And in order to avoid as much as possible the shock which might result from the operation (which is most undoubtedly a severe one,) I invariably rendered the animals insensible, either by the smoke of the common puff-ball or by ether. By so doing, I obtained another advantage, for as the animal remained perfectly still, I was enabled to perform the operation quicker and with much less danger of wounding the neighboring organs. It is perhaps on this account that my operations have been attended with less fatal consequences than those of any other observer.

Brown-Sequard, who I imagine has performed the operati on of the removal of the supra-renal capsules as often as any one else, gives the following table as the result of his experience:

| Number and species of animal. | Average. duration of life. Hours. | Minimum. duration of life. Hours. | Maximum. duration of life. Hours. |
|---|---|---|---|
| 51 Rabits, | 9 | 5½ | 14½ |
| 11 Dogs and cats, (adults) | 14 | 7½ | 17 |
| 2 Mice, | 8 | 7½ | 8½ |
| 11 Guinea-pigs, (adults) | 13 | 9 | 23 |
| 2 " (young) | 23½ | 14 | 33 |
| 11 Young cats and dogs, | 37 | 19 | 49 * |

Here it is seen that the adult animals which he operated upon died on an average in twelve hours, while the young or new-born animals lived thirty hours; and this being the case, we can scarcely be surprised that Dr. Brown-Sequard should have concluded that the supra-renal capsules were more necessary to life than even the kidneys; for animals will live two or even three days after the extirpation of the latter organs.

I am happy to state that since the publication of the above record, Brown-Sequard has continued his researches with more favorable results. He says that of ten rabits from which he extirpated the supra-renal capsules, six died between the seven and tenth hour, and four between the tenth and fourteenth hour after the operation. He adds that these animals died too quickly for it to be possible to attribute their death to peritonitis; further, that the extirpation of the capsules is followed by symptoms which do not occur after injuries of the peritoneum, liver, &c., the symptoms appearing to indicate that the supra-renal bodies have an important influence upon the blood, and that their nerves have a singular power upon certain points of the central nervous system. He thinks that this latter influence manifest itself very distinctly in some cases after the extirpation or the puncture of one of the capsules, the animals being occasionally observed to be seized a few minutes before death by

* Archives Generales de Med., p. 395, 1857.

vertigo, and rolling over. He concludes his remarks by the abservation, first, "that if these organs are not essential to life, they are at least as important as that of the kidneys, for when they are absent, death in general supervenes more rapidly than after the removal of the kidneys."*

To return to my own experiments. The first animal that I operated upon was an adult cat. She lived nine days after the operation. From this animal I removed the right capsule. The operation lasted only ninety seconds. It was accomplished remarkably easily, and without any hæmorrhage.† When the animal became sensible (she had been narcotized by the smoke of the puff-ball,) she seemed to suffer little or no pain. On the day following the operation, the cat took her food well, ann she continued apparantly in good health up till the eighth day. It was then noticed that she had lost flesh. On the ninth day the cat was found dead. On post-mortem examination, the pupils were observed to be fully dilated. The ahdomen appeared entirely free from any signs of inflamation, with the exception of the peritoneal covering of the kidney on the operated side. It was somewhat opaque, and did not permit the superficial vessels to be seen through it as distinctly as that covering the opposite kidney. The immediate case of death could not be ascertained.

As one of my objects in extirpating the capsules was to ascertain if their rcmoval would be attended by any change of color in the skin or its appendages, I took a nearly white cat, and extracted both supra-renal capsules from it at one time. Twenty-four hours after the operation, the animal was found dead, and in a state of *rigor mortis.* In consequence of its being very fat and vicious, it had been kept fasting during nearly four days before the operation was performed. On post-mortem examination, some lymph was found effused upon one of the kidneys. There was no pus, but all the other signs of peritonitis.

On operating upon animals like the cat and dog, I usually found it necessary to ligature a vein of considerable size, which crosses directly over the middle of the capsule; and thinking that the injury done to this vessel might have caused phlebitis, I put a ligature round half of the left supra-renal capsule of a small white coach-dog, avoiding the vein altogether, and cut away the other half of the organ. The next morning, contrary to what might have been expected from the statement of Brown-Sequard, who says that mere puncture of the organ is rapidly fatal, the dog came running to meet me at the door of the room in which he was confined. He seemed to suffer little or no pain, for he frisked about, and

* Archives Generales de Med. p. 374. 1857.

† The attachments of this capsule were unusually slight for a rightne.

was hungry for his food. Six days afterwards, however, he appeared much less lively, and on the ninth day after the operation, he for the first time refused to take any meat. On the tenth he looked very ill, and on the twelfth he died.

*Post-mortem.*—On opening the abdomen, extensive and intense signs of peritonitis were observed. The intestines were glued together, and flakes of lymph abounded all over the abdominal walls. A large abscess, surrounded by an adventitious membrane, two lines in thickness, occupied the left hypochondriac region. It involved the lower half of the spleen. and rested upon the kidney. It did not, however, include the latter organ. In this case there can be no doubt as to the proximate cause of death; but a question might be raised as to whether the inflammation was excited by the injury done to the supra-renal capsule *per se*, or caused by the presence of the ligature, and damage done to the neighboring parts during the operation.

It being a point of primary importance in the prosecution of these researches to ascertain whether the animals died from the injury inflicted upon them by the operation, or solely from the absence of the supra-renal body, and consequent arrest of its function, I proposed taking two animals of the same species, age, sex, and developement—removing a capsule from one, and inflicting upon the other a similar amount of injury, with the single exception of leaving the capsule *in situ*.

My intention was somewhat sooner filled than I had anticipated, for on operating upon a guinea-pig one day, I found that the capsule was traversed, and so intimately attached to a large vein, that it would be impossible to remove it without doing considerable mischief. I therefore left the capsule where it was. and sewed up the wound. I now took the fellow of this guinea-pig, which was of the same sex, age, and development, and removed from him the capsule of the corresponding side.

Both of these animals died within twenty-four hours after the operation. Being somewhat surprised at the result of this experiment, I repeated it upon two cats that had been kept fasting during twenty-four hours before the operation. The cat from which the supra-renal capsule (the right) was removed, lived two days; the other died during the night of the third day. In the abdomens of both, signs of peritonitis were distinctly visible. To prevent mistake, I have to remark that the animal from which the capsule was *not* removed had somethihg more done to it than the mere wound in the abdominal parietes. Had that been the only injury done, it probably would not have died. The experiment would have been incomplete; for, in removing the capsule from the other animal, the neighboring organs, vessels, and nerves could not escape injury; and, independent of the effect arising from the mere absence of the supra-renal capsule, those other mutilations must be taken into account. In order, therefore, to make both operations as nearly as possible alike, with the exception of the removal of the capsule, I handled the kidney, the liver, and the vessels and nerves around the capsule; but so lightly, that I had no anticipation of causing the animal's death. And I must say, that even now I have no idea why it died; for many a time members of the same species of animal have been submitted to much greater mutilations with but a transient deleterious effect, as some of the experiments which shall afterwards be cited amply prove.

Finding that nothing was to be learned by the above course of procedure, except that death might follow the experiment from another cause than mere absence of the capsule, I began to consider whether or not it would be possible to remove the organ in a manner less likely to mutilate the neighboring parts. The supra-renal bodies being very freely supplied by nerves from the solar and renal plexuses, in ligaturing the bundle of vessels it is impossible to avoid including some of the branches of the nerves in the ligature. I thought of giving up using a ligature altogether, and contenting myself with simply twisting the vessels, because I was aware, from the results of experiments upon nerves in other parts of the body, that a ligature applied to them will sometimes induce a greater amount of irritation than the simple division of the same nerve by the knife.

In November, 1856, having obtained a fine, strong bull terrier dog, I resolved to remove one of his capsules without applying a ligature to the vessels. On exposing the left supra-renal body, however, I found it was traversed by a large vein, in such a manner that it could not be removed by the knife without cutting the vessel. It then occurred to me that it might be possible to enucleate the organ with my fingers, and I accordingly attempted to do so; but I found the capsule too friable. It broke to pieces, and I had to remove it portion by portion. Even then I could not get it all away; there were still some small fragments left. In this case there was little or no hæmorrhage; but as the operation lasted some considerable time, I did not expect to find the animal alive the next day. I was agreeably disappointed, however; and although he looked ill, and refused to take food for the first few days after the operation, he gradually recovered, and in a fortnight I had the pleasusure of seeing him running about. This dog remained in my possession until last April, and never was observed to have a single days' illuess, except for a few days when I had cut one of his cervical sympathetics, in order to ascertain the effect of atropine on a permanently-contracted pupil.

The next experiment worthy of notice was one performed upon a large tom-cat. On opening the abdomen of this animal, and touching the right capsule with the point of my finger, I was astonished to find it rough and hard as stone. As it was enucleated with the utmost facility, and without loss of blood, I at once repeated the operation on the left side, with similar success. On making a section of the capsules it was found that a considerable portion of the medullary, as well as of the cortical substance of the organs, had become replaced by a calcareous deposit, which chiefly consisted of carbonate of lime. The remaining portions of the glands contained so much fibrous tissue that the normal structure might be said to have entirely disappeared.*

This case was pregnant with interest to me. Here I had an animal from which nature might be said to have removed the supra-renal bodies; for they were in such an advanced state of disease, that their function, whatever it might be, must have been entirely interrupted. I could therefore study the effects of absence of the organs without the interference of the complicated effects of the operation. The animal at the time of the operation was in excellent health, fat and strong: a most important fact when it is remembered that Dr. Addison supposes that derangement in the function of the supra-renal capsules is accompanied by extreme emaciation and debility. As patients rarely die of supra-renal capsular disease alone, it may yet be a question whether we are right in attributing the emaciation and weakness to the disease of these organs. For here, in the above case, where the capsules were the only organs diseased, the animal's health, if I may judge from the violent display he made of his physical powers, was not in any way deteriorated.

Judging from the size of the supra-renal bodies, I should imagine that the calcareous degeneration did not attack them until after the animal had reached the adult period of life; but whether or not the color of its hair had become changed as the disease advanced, I had no means of ascertaining. The cat, which was of a tabby colour, besides being a valuable subject on account of its enabling me to study the effects of absence of the supra-renal capsular functions without any other complication, was equally interesting in permitting me to ascertain the true amount of injury done in removing the capsules, independent of the arrest of their function—a point which it was impossible justly to appreciate in extirpating healthy organs.

As the operation on this animal had been performed with the utmost

* These supra-renal capsules were shown to the Pathological Society in the month of February last.

facility, and there had been no hæmorrhage, or any apparent injury done to the surrounding parts, except the tearing through the nerves, I felt sanguine of success. My astonishment was therefore great when, arriving at the college next morning, I found the cat dead. What could he have died from? It could not be from the absence of the supra-renal capsular function, for he had lived and thrived after the organs were too diseased to function. It was not from loss of blood, as there had been no hæmorrhage. It could not be from the direct shock of the operation, as it was performed while he was insensible. It was not on account of the mere wound in the abdominal parietes, for laying open the abdomen, and making even a hernia with the intestines, is not sufficient to kill a cat. What, then, could be the cause of death? Will the post-mortem appearances in the abdomen reveal it? Unfortunately they did not.—Nothing, absolutely nothing, was to be seen but a very trifling attempt at peritonitis. The thorax was searched; the brain and spinal cord were examined; the blood vessels were laid open; yet no clue to the cause of death could be detected.

What parts of the body had received injury during the operation besides the abdominal walls, the effects of which we may entirely dismiss from our attention? In removing the capsules their blood vessels, lymphatics, and nerves had been torn across. We had need to dwell long upon the probable effects resulting from the damage done to the two former, for both the blood vessels and lymphatics were insignificant, in consequence of the capsules being in great part a hard mass of inorganic matter. The blood was searched for signs of phlebitis, with a negative result. It was examined for crystals, without more than the usual quantity being detected;* for the flakes of pigment described by Brown-Séquard as blocking up the central capillaries, and none were found.

† Dr. Brown-Sequard, in his memoir on the supra-renal capsules, says, that in order to obtain crystals from normal blood, it is necessary to use ammonia or some other reagent. This statement must be taken with a certain degree of reserve, for during the last half-dozen years I, like many others, have been in the habit of obtaining crystals from normal blood by simply allowing a drop to dry on a slipof glass, then adding a drop of distilled water, covering it up with a piece of thin glass, and setting it aside to crystalize. Sometimes the crystals are large, and form rapidly, at other times they are small, and occasionally altogether absent; this I imagine depends upon the state of the digestion of the animal from which the blood was drawn. They are sometimes colored, sometimes colorless. Crystals rapidly form in the blood of starved animals, or those that have been subjected to any severe operation. They are usually more easily obtained from the blood of the spleen than that drawn from the general circulation. To those of my readers who take interest in this subject, I would strongly recommend the perusal of an able review on blood crystalization, containing much original matter, by Dr. Sieveking, in the twelfth vol. of this Journal.

The lymphatics were not specially examined. It now remains for us to glance at the probable effects arising from the tearing across of the nerves going to the supra-renal capsulus; these, although small, "are exceedingly numerous. They are derived from the solar plexus of the sympathetic, and from the renal plexuses. According to Bergmann, some filaments come from the phrenic and pneumogastric nerves. They are made up mainly of dark-bordered white fibres, of different sizes, and they have many small ganglia upon them."*

As neither the microscope nor chemical reagents can here much assist us, we must call expermental physiology to our aid, and see what we can discover by reasoning from analogy. The question is what would be the result of mutilating the above-named nerves, independent of the removal of the supra-renal capsules? It is an established fact, that instant death frequently follows sudden injury to the solar plexus. A blow on the epigastrium is often sufficient to prove rapidly fatal to life.

Section of the great splanchnic nerves has been found by Ludwig and Haffer to kill animals in the space of one or two days. The mere pricking or cutting of the semi-lunar ganglion, according to Brown, proves fatal in rabbits in the course of thirty hours; while division of the sympathetic in the neighborhood of the kidney causes death within twenty-three hours after the operation. Such being the case, need we hesitate, in the absence of all other proof as to the cause of death in the above-mentioned experiment, to regard it as probable that the animal died from the injury done, in tearing out the capsules, to the ganglionic system of nerves? I shall again have occasion to recur to this subject.

Having now given my readers a general view of the manner in which the experiments were conducted, and the results obtained, I shall refrain from citing any except those which present the subject of inquiry in a special and important light.

Is the extirpation of the right a more fatal operation than the removal of the left capsule? This question I think I may answer in the affirmative. In proof of this I cannot do better than relate the following experiments:—From two healthy cats I removed the right supra-renal capsules. The one animal died in forty, the other in forty-four hours after the operation. The only symptoms which they presented were those of gradually increasing weakness. On post-mortem examination the abdomens of both showed slight signs of peritonitis.

From two other cats, which could scarcely be regarded as so healthy as the preceeding, in consequence of my having a few days previously divi-

* Quain's Anatomy, edited by Professors Sharpey and Ellis, vol. iii. p. 331.

ded one of their cervical sympathetic nerves, I extracted the left supra-renal capsules. In one of these animals an abscess formed in the vicinity of the wound, and burst externally on the fourth day after the operation; after which he gradually sank, and died on the eighth day. On opening the abdomen slight signs of peritonitis were found. The internal wound was nicely filled up by omentum, external wound nearly completely healed. The other cat never had a bad symptom; and thirty days after the operation he looked so strong and healthy that I resolved to remove also the right capsule.

The results of these four experiments, and of several others which I need not cite, I think authorize me to have answered the question at the head of this chapter in the affirmative. Were it necessary, I might relate many others, to prove, that not only in cats, but also in all the different species of animals on which I operated, the removal of the right was, as a general rule, a more fatal operation than the removal of the left supra-renal body. Allowing this statement, then to be a fact, the next question is, why should the extirpation of the right prove so much more fatal than that of the left capsule? M. Gratiolet, whose experiments led him to the same conclusion, thought that it depended upon the proximity of the liver, and the injury received by that organ during the operation, giving rise to hepatitis. At first I was of a somewhat similar opinion; but after seeing several cases of rapid death follow the operation, where neither hepatitis nor even marked peritonitis existed, I felt forced to look for some other cause. A close examination of the two capsules soon showed me that the difference in fatality of the operations could scarcely be said to exist in the function of the one being more important than that of the other capsule; the organs being identical in structure, and nearly of equal size. Perhaps the right may in some cases be a little larger than the left, but this difference is too inconsiderable to account for the great difference in the fatality of the two operations. On studying the anatomical relations of the supra-renal bodies, it was easy to see that the right was much more intimately connected with the surrounding parts than the left, and its removal attended with greater difficulty. The former lies higher in the abdomen than the latter; it is in contact above with the liver, in front with the vena cava, below with the upper margin of the kidney; and behind it in most cases touches the right semilunar ganglion. The left supra-renal body lies farther from the mesial line; it is in contact below with the upper margin of the kidney, in front (usually) crossed by a branch of the vena cava, behind with the parietes of the abdomen, and above with the spleen, but not adherent to it.

The right semilunar ganglion, which is much larger than the left, lies (in the dog and cat) directly beneath the supra-renal capsule; while the smaller left semilunar ganglion is placed on the left crus of the diaphragm, and internal to the supra-renal body. A glance at these anatomical relations shows that much greater injury is likely to be done to the sympathetic system of nerves in the extirpation of the right than of the left supra-renal capsule. And from what has already been said with regard to the immediate cause of death, probably arising from the mischief done to the ganglions, it is easy to understand why the removal of the right should, as a general rule, prove more rapidly fatal than that of the left supra-renal body.

In the case of double organs, it is usually found that after the removal of one, the other becomes after a time somewhat enlarged, from having taken on the functions of its fellow in addition to its own. I was anxious to see if the same result had occurred in the case of the cat which made such a good recovery after the removal of the left supra-renal capsule. On careful examination, it was found neither inflamed nor hypertrophied; but it must be remembered that only a month had elapsed since the removal of the other capsule. After this latter operation, the animal cried for some hours, as suffering extreme pain. The next day it appeared very much depressed, and on the morning of the third day it was found dead, with the pupils dilated, and in a state of well-marked *rigor mortis.*

On examining the abdomen, signs of peritonitis were seen in the neighborhood of the right kidney, the capsule of which was thickened and opaque. In the wound behind the kidney was a considerable quantity of pus. The great abdominal and thoracic veins were engorged with partly-coagulated blood. The right side of the heart was full of purple-colored blood, on some of which being poured over the surface of a white vessel, it presented that granular look usually seen in blood beginning to putrify. It did not oxygenate readily on exposure to the air. When examined with the microscope, a great number of lymph globules were found in it. No crystals could be obtained from either the liquid or coagulated blood. No signs of inflammation were detected in the large veins. Two of the lumbar lymphatic glands were very much enlarged, especially the one on the right side of the abdomen. It presented a peculiarly beautiful, semitransparent, lobular appearance, such as I had never before witnessed.

The gland was placed in a little water preliminary to preserving it; but the next day it was found to have lost its strange lobular appearance, and looked like an ordinary lymphatic gland. The hypertrophied gland on the left side of the abdomen, on being examined with the microscope, was found full of well-formed circular lymph cells.

I tried to removed the supra-renal capsules from white mice, but I found them too delicate to stand the operation. With rats, on the other hand, I was more fortunate. The rat, indeed, is admirably well suited for this experiment, in consequence of the attachments of its supra-renal bodies being extremely slight. The organ on the left side hangs almost loosely in the abdomen; its vessels and nerves are nearly its only bands of union. On the right side, again, it is somewhat more intimately connected with the neighboring parts, but not sufficiently to give rise to any great difficulty in its removal. The supra-renal capsule of the rat is about the size of a pea, and of a circular form, in consequence of its very slight attachments.*

The following is the result of one of my first experiments upon a white rat. The animal having been rendered insensible, I made an incision on the right side of the abdomen, close under the floating ribs, and removed the supra-renal capsules. Next day, the respirations were 139 in the minute. The animal, however, rapidly recovered, and nine days afterwards it appeared so well, that I removed the left capsule also. The latter came very readily away,—neither knives nor ligatures were required. A single twist with the forceps at once removed it, and without hæmorrhage.

The color of the hair and of the skin was carefully watched without any peculiarity being observed until about ten days after the last operation, when the neck of the animal was noticed to have become denuded of hair: the skin, however, retained its white color. The animal began from this time to refuse food, and seemed gradually to get weaker and weaker, until it died, twenty-five days after the extirpation of the right, and sixteen days after the removal of the left supra-renal capsule. The exterior of the body was carefully examined, but no discoloration of the skin could detected. The portion of the skin that had been denuded of hair was now seen to be covered with a luxuriant crop of young hair, about one-eighth of an inch long, showing that the affection of the cutaneous appendages had not been permanent, but merely transient. It is not at all uncommon for animals to lose part of their hair after severe operations. I have seen this very frequently in rabbits and cats.

On opening the abdomen of this rat, the intestines looked like a gela-

---

* *Vide* the researches of Professor Ludwig Fick on the cause of the form of bones. Gottingen, 1857.

tinous mass: at the first glance it was impossible to recognise what they were, and it was only on close examination that they were found to be the intestines. Their walls were so thin and transparent that the air-bubbles could be distinctly seen floating in their interior.* The left half of the stomach was healthy, but the right half was in a similar condition to the intestines. It contained a clear amber-colored mucus with a faintly alkaline reaction. This proved that the softening was not produced by the post-mortem action of the gastric juice. A similar condition of the stomach and intestines has been often observed in ill-fed, half-starved children, and more especially in those cases where they die of a depressed condition of the ganglionic nervous system. I believe that the degeneration of the stomach and intestines of this animal may have had for its cause the injury done to the ganglionic system of nerves, especially those branches supplied to the capsules by the solar plexus. The left capsule, as was said, had been extracted with the greatest facility, and on that side the wound in the peritoneum could only be detected on the closest inspection. By the mere examination of the internal parts no one could have imagined that such an operation had been so lately performed.

The removal of the right capsule had been attended with some difficulty, in consequence of its closer adherance to the inferior vena cava and neighboring parts. On this side the margin of the liver was found attached to the wound by somewhat strong adhesions. The right side of the stomach only being in a state of gelatiniform degeneration might be owing to the greater injury done to the nerves on that side, for the anatomical reasons before mentioned.

Other experiments on the rat proved more successful. I shall quote the following as it has a peculiar interest on more than one account.

In March last I removed the right supra-renal capsule from a piebald rat. The operation was a very disagreeable one, in consequence of the animal not having been rendered sufficiently insensible. As it lasted several minutes I had little hope of the animal's surviving. Contrary to my anticipations, however, he made a tolerably rapid recovery, and in the beginning of May, as he was in excellent condition, I removed the other capsule. As six weeks had elapsed since the extirpation of the right supra-renal capsule, I carefully examined the left, and compared it with other capsules taken from animals of the same species and age, in order to ascertain if it had become hypertrophied. But in this, as in the case of the cat, I could not find any very marked difference in either its

* The intestines of mice and rats are usually somewhat transparent, but nothing in comparison to what was here observed.

size or appearance. From the effects of the latter operation the animal speedily recovered, and he ultimately (in the course of a month or two) became very fat and healthy-looking. I think, after reading the report of this case, one will scarcely be inclined to assert that the supra-renal capsules are absolutely essential to life, or that the absence of their function is attended with emaciation and debility. I know that some will say that the function of the supra-renal capsules had been in this case vicariously performed by another organ. To these gentlemen I have only to reply that I do not deny this; but that their argument is equally applicable to the human subject, and if they apply it in the one, they ought to apply in the other case also.

With regard to the connexion of the supra-renal capsular function and bronzing of the skin, I shall at present merely remark, that in the case of this rat no increase of pigment was observed to take place. One day I was told by a gentleman who occasionally came to see my animals without supra-renal capsules, that the fact of no deposit of pigment ooccurring in the skin, or its appendages, of white rats after the removal of their capsules, was no argument against the idea that the function of the supra-renal bodies was so to modify the animal coloring matter as to prevent its becoming deposited in the skin. For, said he, the peculiar constitution of the albino is exactly such as to prevent any deposition of pigment taking place. I would bring forward several arguments against this view, were it not that they are rendered entirely unnecessary by the result obtained in this case. The objection raised by the gentleman against the albinoes does not hold good with regard to the piebald animal. Being half brown, it could not have possessed any peculiar idiosyncrasy preventing a deposit of pigment. Yet, not withstanding that it was without the organs supposed to be essential to the transformation of pigment during a quarter of a year,* no increased deposit either in the skin or its appendages could, after the most searching scrutiny, be detected. What are we to conclude from this fact? Is the theory of bronzed skin untenable in the case of certain animals?

* One hundred and twenty days after the removal of the right, and eighty days after the extirpation of the left supra-renal capsule, I took away the spleen from this animal. The blood of the spleen immediately after the operation was examined by the gentlemen attending my practical class, and found to contain an immense number of white corpuscles. Only one of the gentlemen, Mr. Mauley, obtained it crystalized. His specimen was full of beautiful rhombic prisms and needle-shaded crystals. The animal died a fortnight after the latter operation. On opening the abdomen, the stomach and part of the intestines were found adherent to the recent wound, while the liver and another part of the intestines were attached to the parts surronnding the old wound on the right side. No supra-renal capsules were found.

Rats not only survive after extirpation of the supra-renal capsules, but even become strong and healthy after the removal of the spleen also. I have at present in my possession some animals from which the spleen and supra-renal capsules were removed when they were a month old. Yet, notwithstanding this, they have grown up without any apparent modification of their functions having taken place, and are now fine specimens of the well-developed adult animal.

Monsieur Philipeaux has even been more successful; he has removed the spleen, the supra-renal capsules, and the thyroid glands from the same animal, and it has afterwards recovered. I removed the thyroids from a rat nine weeks after the removal of its spleen and supra-renal capsules, but the animal died two days after the operation. Its blood crystalized with the utmost facility. The crystals were almost all colorless. In the urine of this animal I found a great number of large bundles of needle-shaped crystals. They appeared to be either hippuric, or a peculiar form of uric acid, but unfortunately I had not a sufficient quantity to ascertain their reactions

While in Paris last summer, Monsieur Philipeaux—whose experiments have led him to the same conclusions as myself—and I repeated our experiments together on a number of young rats, some of which I brought to England with me; and although it is now more than a quarter of a year since their spleens and supra-renal capsules were extirpated, the animals are still alive and well. I intend to breed from them, in order to see if, after the third or fourth generation of animals without spleen and supra-renal capsules, any change in the cutaneous or other functions will be observable. One of the rats that I brought home with me was the offspring of a mother without either the supra-renal capsules or spleen. From it Monsieur Philipeax extracted those organs when it was aged twenty-six days; it is now four months old, and although scarcely so large as the generality of animals of the same species at that age, is yet quite healthy and strong.

From the results of the foregoing experiments, I think we may draw the following conclusions:—

1st. The supra-renal capsules are not solely fœtal organs.

2d. The supra-renal capsules are not absolutely necessary to life.

3d. The removal of the right is generally more fatal than removal of the left capsule.

4th. That convulsions do not necessarily follow the removal of the capsules.*

---

* In a letter dated November 15th, Professor Virchow informs me that he has

5th. That the absence of their function, (in rats) is attended neither by great emaciation nor debility.

6th. That when death follows npon the extirpation of the supra-renal bodies, it is in most cases in consequence of the injury done to the neighboring tissues ; perhaps, most frequently the mutilation of the ganglionic system of nerves.

7th. Absence of the function of the supra-renal bodies is not proved to have any special effect in arresting the transformation of hæmatin, or in increasing the formation of blood crystals.

8th. The suppression of the supra-renal capsular function is not at_ tended by an increased deposit of pigment in the skin or its appendages (in rats.)

9th. The problem of the connexion of bronzed skin and supra-renal capsular disease is more likely to be solved in the dead house than in the physiological laboratory.

*To be Continued.*

